# Mental health literacy among secondary school learners in Tshwane region 1: A quantitative study

**DOI:** 10.4102/sajpsychiatry.v31i0.2349

**Published:** 2025-04-10

**Authors:** Dumisile P. Madlala, Pierre Joubert, Oratilwe P. Mokoena

**Affiliations:** 1 Department of Health, Faculty of Science, Tshwane District mental health services, Tshwane, South Africa; 2 Department of Health, Faculty of Science, University of Pretoria, Tshwane, South Africa; 3 Department of Statistics, Faculty of Sciences, Sefako Makgatho University, Tshwane, South Africa

**Keywords:** mental health literacy, learners mental health literacy, school learners, Tshwane, South Africa

## Abstract

**Background:**

Mental health literacy (MHL) is one of the crucial factors in the prevention and maintenance of youth mental health. Despite this fact, there is limited research on MHL in this age group.

**Aim:**

To determine the MHL in a sample of secondary schoolgoing learners.

**Setting:**

Five schools in Tshwane, South Africa.

**Methods:**

A quantitative cross-sectional study was done. Three fictive vignettes depicting individuals having symptoms of major depressive disorder (MDD), substance-induced psychotic disorder (SIPD) and social anxiety disorder (SAD) were presented to participants. The ability to recognise the disorder, knowledge of the best form of help to address the symptoms, and the ability to provide psychological first aid support were assessed. A comparison of MHL between township school learners and urban school learners was conducted. The association between MHL and demographic factors was also assessed.

**Results:**

The ability to recognise symptoms and connect them to a particular disorder was high (80.71% for MDD, 61.96% for SIPD and 67.91% for SAD). Correct knowledge on who would best address the symptoms was 52.55% for MDD, 63.83% for SIPD and 23.86% for SAD with a sizable number choosing informal help for the cases of MDD and SAD. There was good psychological first aid knowledge for both MDD and SIPD cases but poor for SAD case.

**Conclusion:**

Even though the results are promising regarding the recognition of all three disorders, there is still room for improving MHL in this group, especially in the areas of help-seeking and knowledge about anxiety disorders in general.

**Contribution:**

The findings highlight key areas of focus during mental health awareness campaigns to learners.

## Introduction

Mental health-related problems have been found to affect about 20% of young people worldwide.^1^ Those affected have a higher prevalence of deleterious outcomes like suicide attempts and completion, negative social and psychological development,^2,3^ and reduced productivity and poor quality of life.^3^ Evidence also indicates that the likelihood of such negative outcomes is less after contact with mental healthcare services.^4^ One study even showed up to 60% of people benefiting from early contact with services.^5^

Having contact with mental healthcare professionals depends on factors like knowledge about available services, the ability to recognise symptoms that warrant consultation, and having an attitude that promotes help-seeking behaviors.^6,7,8^ Therefore, raising awareness and improving literacy on mental health-related issues in young people has the potential to improve and maintain general mental well-being. Mental health literacy (MHL) is considered one of the major determinants of mental health outcomes.^8,9,10^ It can lead to the early identification and treatment of mental disorders and contributes towards professional help seeking and the use of mental health services.^11^

Mental health literacy is defined as knowledge and attitudes that promote mental well-being.^12^ There are four main domains of MHL: (1) understanding how to obtain and maintain positive mental health; (2) understanding mental disorders and their treatments, including the ability to recognise symptoms of a mental disorder, the knowledge of best help available and the knowledge about the risks factors for mental disorders; (3) decreasing stigma related to mental disorders; and (4) enhancing help-seeking efficacy.^13^ Knowledge about mental disorders is important because knowing how to identify symptoms of a particular disorder alerts the individual who is experiencing symptoms that the experiences may need professional attention. On the other hand, knowledge about the best form of help available may encourage early help-seeking behaviors.^14,15^

Despite the evidence on the potential good that raising MHL can provide for the youth, there is limited research on MHL in this age group compared to adults worldwide, including in South Africa.^13^

South Africa is an upper middle-income country with about 60 million residents.^16^ According to the statistical report, 22% of the 60 million residents are between ages 10 and 19 years.^16^ South Africans face much social stressors such as high unemployment rate, gender-based violence, racial discrimination, etc. These stressors are associated with an increased prevalence of mental disorders.^17^ Young people and children in South Africa are not exempted from experiencing these stressors and subsequently, mental health-related problems. It is estimated that 17% of people under the age of 19 years in South Africa suffer from a mental disorder.^18^ Another study also reported the likelihood of developing mental disorders to be about 15%, specifically in learners.^19^ As it is, this evidence indicates that young people in South Africa are at higher risk of developing mental health problems. If this is the case, and MHL is one of the tools that can be used to combat the mental illnesses, it is imperative to question and seek to know the level of MHL of young South Africans. As a result, this study is done.

### Study aim

The purpose of this study is to determine the MHL in secondary schoolgoing learners in the City of Tshwane, Gauteng Province, South Africa.

### Objectives

- To determine the learners’ ability to recognise three mental disorders, namely, major depressive disorder (MDD), social anxiety disorder (SAD) and substance-induced psychotic disorder (SIPD).
- To determine the learners’ knowledge about available professional help.
- To assess the learners’ knowledge about how to give psychological support to other learners.
- To compare the level of MHL literacy between the township school learners and urban school learners.
- To compare the literacy level in relation to sociodemographic factors (i.e. the gender, marital status of the parents and employment status of the parents).

## Research methods and design

### Study design

A quantitative cross-sectional descriptive study was carried out between July 2023 and September 2023.

### Study site

**FIGURE 1:**
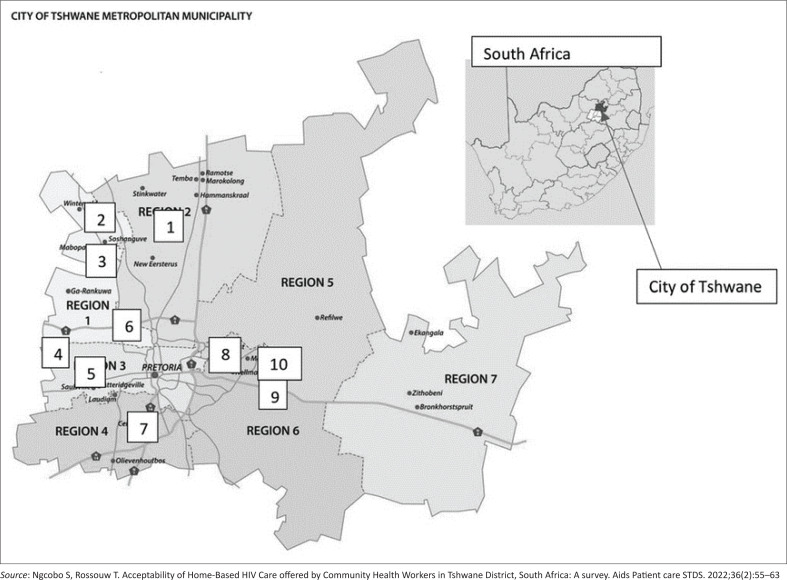
The regions of Tshwane.

The study was conducted in the City of Tshwane, which is divided into seven regions (Figure 1^20^). The City of Tshwane has a population of approximately 3 million people.^21^

Region 1 has five subregions; it was conveniently sampled from a total of seven regions. It has a population of 811 570^21^ with 59 secondary schools.^22^ In each of the five sampled subregions, one secondary school was randomly selected. The townships where the schools were sampled were the areas of Mabopane, Soshanguve, Winterveld and Ga-Rankuwa, and the urban area was Pretoria North.

### Study population

The study population were secondary schoolgoing consenting males and females between the ages of 13 and 19 years.

### Sample size and sampling technique

The sample size was calculated by using registered software Raosoft^®^ using the following assumptions: 4918 populations; 50% proportion; 95% confidence interval; 5% margin of error, design effect of 1.5 and a non-response rate of 10%; and the minimum sample size required for the study is 529. The sample size was allocated with a probability proportionate to the size of the schools. In each school, two classes for each grade (i.e. 8–12) were randomly selected from the list provided by the principal. A total of a convenient sample of 10 classes in each school and a minimum of 10 students were consecutively selected per class.

### How was the study conducted?

The study information sheet and the consent form were presented to learners to take home to their parents or guardians to read and sign if they would agree that their children may participate in the study. Once the parents had signed, the school health nurses or school authorities informed the first researcher of the number of the consent forms returned. A subsequent suitable date and time was set for the researcher to come back to the school and conduct the research. On the day, the assent forms were first distributed to the learners. A thorough explanation was provided, including confidentiality and the right to abstain from participating if not willing to participate. They were also informed to read through the form and ask any questions if some information was not clear. Because the vignettes might have had the capacity to upset sensitive learners, the researcher provided extensive information to participants and school authorities on how participants may access mental healthcare services should the need arise. The information included a pamphlet listing common websites with information about the conditions described in the vignettes (and other mental health information).

### Data collection

The questionnaire consists of two parts, Section A and Section B. Section A includes sociodemographics that provide information about the learners and their parents. Section B contains three fictive vignettes of people presenting with symptoms of three different mental disorders, followed by questions related to the vignettes.

The vignettes meet the DSM-5 criteria for MDD (the case of Betty), SAD (the case of Lucy) and SIPD (the case of Muzzy). These disorders were selected because they are considered common in South Africa.^23^ Participants were asked three questions with possible answers in multiple-choice format. To determine the ability to recognise the mental disorders, participants were asked what type of illness they think each person is suffering from, and to assess knowledge of the best form of treatment, they were asked what the best form of treatment for each of the people in the vignettes is. To assess knowledge of psychological first aid support, they were asked about what they would do to help the person in the vignette if the person were a friend. The correct answer to each question is coded as 1 for a correct answer; otherwise, it is coded as 0.

### Statistical analysis

Data were captured using Microsoft Excel and exported to the Statistical Package for Social Science (SPSS) software version 30^24^ for analysis. The results were summarised using frequencies and percentages for categorical variables and mean and standard deviation for continuous variables. Chi-square and independent student *t*-tests were applied for comparative analysis between groups for categorical and continuous variables, respectively. A *p*-value of less than 0.05 was considered statistically significant.

### Ethical considerations

Ethical clearance to conduct this study was obtained from the University of Pretoria Faculty of Health Sciences Research Ethics Committee (No. 5/2023) and the Department of Education. All participants and their parents or guardians were informed of the voluntary nature of their participation. For learners aged 18 years and younger, parents were requested to complete an informed consent form on their behalf. The learners were also given an assent form to read and understand the study before deciding whether they wanted to participate. Permission was requested from the relevant school authorities that were randomly selected.

## Results

The demographics are displayed in Table 1. A total of 529 questionnaires were distributed to five different schools. Each participant had to respond to three vignettes describing three scenarios, which were MDD, SIPD and SAD. The results obtained from participants’ chosen answers are displayed in Tables 2 to 4. The descriptive statistics comparing township and urban schools regarding recognising the mental disorder, the best form of help, and how to assist are displayed in Table 5. Lastly, the descriptive statistics comparing the ability to recognise the mental disorder in relation to the demographic information are displayed in Table 6.

**TABLE 1:** Demographics (*N* = 529).

Variables	*n*	%
**Gender**		
Male	155	29.30
Female	374	70.70
**Age (years)**		
13–17	460	86.96
18–21	69	13.04
**Current grade**		
8	146	27.60
9	79	14.93
10	140	26.47
11	136	25.71
12	28	5.29
**Current living conditions**		
Stay with both parents	237	44.80
Stay with one parent	216	40.83
Stay with an adult guardian	61	11.53
Child headed family – guardian is an older sibling	15	2.84
**Employment status of parent or guardian**		
Unemployed	199	37.62
Part-time employed	69	13.04
Full-time employed	261	49.34

**TABLE 2:** Participants’ responses regarding the recognition of a mental disorder.

Options	MDD (Betty) *N* = 425		SIPD (Muzzy) *N* = 439		SAD (Lucy) *N* = 455	
	*n*	%	*n*	%	*n*	%
(a) Physical illness (an illness of the body)	19	4.47	13	2.96	22	4.84
(b) Mental illness (an illness of the mind)	44	10.35	74	16.86	43	9.45
(c) Depression	343	80.71	22	5.01	25	5.49
(d) Social anxiety disorder	12	2.82	45	10.25	309	67.91
(e) Substance-induced psychotic disorder	4	0.94	272	61.96	43	9.45
(f) I don’t know	3	0.71	13	2.96	13	2.86

MDD, major depressive disorder; SIPD, substance-induced psychotic disorder; SAD, social anxiety disorder.

**TABLE 3:** Participants responses regarding who can best help the people in the vignette.

Options	MDD (Betty) *N* = 451		SIPD (Muzzy) *N* = 412		SAD (Lucy) *N* = 415	
	*n*	%	*n*	%	*n*	%
(a) They can deal with the symptoms alone	18	3.99	15	3.64	0	0.00
(b) Close friends and close family members	110	24.39	1	0.24	175	42.17
(c) Teacher	21	4.66	16	3.88	60	14.46
(d) Non-specialist mental health practitioners like family doctors or clinic doctors	56	12.42	104	25.24	61	14.70
(e) Specialists’ mental health practitioners like psychiatrist, psychologist and social workers	237	52.55	263	63.83	99	23.86
(f) I don’t know	9	2.00	13	3.16	20	4.82

MDD, major depressive disorder; SIPD, substance-induced psychotic disorder; SAD, social anxiety disorder.

**TABLE 4:** Participants’ responses on how they can best assist each of the people in the vignette.

Options	MDD (Betty) *N* = 478		SIPD (Muzzy) *N* = 473		SAD (Lucy) *N* = 446	
	*n*	%	*n*	%	*n*	%
(a) Not offer any help as you are allowing them their privacy	22	4.29	10	2.11	0	0.00
(b) Tell them to keep it a secret as many other people will avoid them	22	4.29	28	5.92	25	5.61
(c) Advise them to use drugs to calm the mind	18	3.51	36	7.61	22	4.93
(d) Rally other friends to cheer them up	75	14.60	33	6.98	187	41.93
(e) Listen to them in an understanding way and suggest they seek professional help	325	63.30	327	69.13	179	40.13
(f) I don’t know	16	3.12	39	8.25	33	7.40

MDD, major depressive disorder; SIPD, substance-induced psychotic disorder; SAD, social anxiety disorder.

**TABLE 5:** Comparison between the urban school and township schools regarding recognizing the mental disorder, best form of help and how to assist.

Variables	Township schools		Urban school		*p*-value at 95% CI
	Correct response (*n*)	%	Correct response (*n*)	%	
**Recognition**					
MDD	36	9.92	10	6.25	0.172
Psychosis because of substance	173	48.87	99	62.66	0.004*
SAD	185	52.56	124	77.99	< 0.001*
**Best form of help**					
MDD	153	42.74	84	52.50	0.039*
Psychosis because of substance	172	49.14	91	57.96	0.066
SAD	63	17.85	36	22.64	0.204
**How can you assist**					
MDD	224	63.46	101	63.13	0.943
Psychosis because of substance	207	59.14	120	75.00	< 0.001*
SAD	113	32.85	66	41.51	0.059

* Indicates findings that are statistically significant.

MDD, major depressive disorder; SAD, social anxiety disorder; Cl, confidence interval.

**TABLE 6:** Comparison between ability to recognise the mental disorder and demographic factors.

Variables	MDD (Betty)					SIPD (Muzzy)					SAD (Lucy)				
	Incorrect		Correct		*p*	Incorrect		Correct		*p*	Incorrect		Correct		*p*
	*n*	%	*n*	%		*n*	%	*n*	%		*n*	%	*n*	%	
**Gender**															
Male	46	29.68	109	70.32	0.089	71	46.10	83	53.90	0.538	70	45.45	84	54.55	0.217
Female	140	37.43	234	62.57	-	182	49.06	189	50.94	-	147	36.62	224	60.38	-
**Age (years)**															
13–17	158	34.35	302	65.65	0.312	222	48.68	234	51.32	0.561	191	41.89	265	57.61	0.509
18–21	28	40.58	41	59.42	-	31	44.93	38	55.07	-	26	37.68	43	62.32	-
**Current grade**															
8	50	34.25	96	65.75	0.607	76	53.52	66	46.48	0.038*	68	47.89	74	52.11	0.108
9	22	27.85	57	72.15	-	43	54.43	36	45.57	-	34	43.04	45	56.96	-
10	53	37.86	87	62.14	-	68	48.57	72	51.43	-	55	39.29	85	60.71	-
11	50	36.76	86	63.24	-	59	43.38	77	56.62	-	54	39.71	82	60.29	-
12	11	39.29	17	60.71	-	7	25.00	21	75.00	-	6	21.43	22	78.57	-
**Current living conditions**															
Child headed family – guardian is an older sibling	6	40.00	9	60.00	0.055	11	73.33	4	26.67	0.017*	10	66.67	5	33.33	0.005*
Stay with an adult guardian	20	32.79	41	67.21	-	26	42.33	34	56.67	-	28	46.67	32	53.33	-
Stay with both parents	70	29.54	167	70.46	-	100	42.55	135	57.45	-	79	33.62	156	66.38	-
Stay with one parent	90	41.67	126	58.33	-	116	53.95	99	46.05	-	100	46.51	115	53.49	-
**Employment status of parent or guardian**															
Full-time employed	67	25.67	194	74.33	< 0.001*	98	38.13	159	61.87	< 0.001*	76	29.57	181	70.43	<0.001*
Part-time employed	26	37.68	43	63.32	-	42	60.87	27	39.13	-	31	44.93	38	55.07	-
Unemployed	93	46.73	106	53.27	-	113	56.78	86	42.22	-	110	55.28	89	44.72	-

* Indicates findings that are statistically significant.

MDD, major depressive disorder; SIPD, substance-induced psychotic disorder; SAD, social anxiety disorder.

### On recognition of a mental disorder

Not all learners completed the questions related to all three vignettes, or all the sections of the individual vignettes, but the numbers are nonetheless enough for statistical analysis. The correct answers for the vignettes are Betty: mental disorder and depression; Muzzy: mental disorder and SIPD; and Lucy: mental disorder and SAD.

The MDD vignette was answered by 425 participants. The correct answer, depression, was chosen by 343 (80.71%) participants, but only 44 (10.35%) identified it correctly as a mental disorder. Incorrect choices were physical illness (19, 4.47%), social anxiety (12, 2.82%), SIPD (4, 0.94%) and I don’t know (4, 0.71%).

The SIPD vignette was answered by 439 learners. The correct choice, SIPD, was chosen by 272 (61.96%) participants, and 74 (16.86%) identified it correctly as a mental disorder. Incorrect choices were SAD (45, 10.25%), physical illness (13, 2.96%) and I don’t know (13, 2.96%).

The vignette of SAD was answered by 455 learners. The correct choice, SAD, was chosen by 309 (67.91%) participants, but only 43 (9.45%) identified it correctly as a mental disorder. Incorrect choices were physical illness (22, 4.84%), depression (25, 5.49%), SIPD (43, 9.45%) and I don’t know (13, 2.86%).

### On whom can best help in each vignette?

The correct answers for all vignettes are non-specialist mental healthcare practitioners like family doctors and clinic doctors and specialists’ mental healthcare practitioners including psychiatrist, psychologist and social workers.

The MDD vignette was answered by 451 participants. The correct answers, specialists’ mental healthcare practitioners and non-specialists’ mental healthcare practitioners, were chosen by 237 (52.55%) and 56 (12.42%) participants, respectively. Incorrect choices were: they can deal with the symptoms alone (18, 3.99%), close friends and close family members (110, 24.39%), teachers (21, 4.66%) and I don’t know (9, 2%).

The SIPD vignette was answered by 412 participants. The correct choices – specialists’ mental healthcare practitioners and non-specialists’ mental healthcare practitioners – were chosen by 263 (63.83%) and 104 (25.24%) participants, respectively. Incorrect choices were: they can deal with the symptoms alone (15, 3.64%), close friends and close family members (1, 0.24%), teachers (16, 3.88%) and I don’t know (13, 3.16%).

The SAD vignette was answered by 415 participants. The correct answers – specialists’ mental healthcare practitioners and non-specialists’ mental healthcare practitioners – were chosen by 99 (23.86%) and 61 (14.70%) participants, respectively. Incorrect choices were: they can deal with the symptoms alone (0, 0%), close friends and close family members (175, 42.17%), teachers (60, 14.46%) and I don’t know (20, 4.82%).

### On how best you can assist each of the people in the vignette

For all three vignettes, the most appropriate answer to the question was to listen to them in an understanding way and suggest they seek professional help.

The MDD vignette was answered by 478 participants. The correct answer was chosen by 325 (63.3%) participants. Incorrect choices were: not offering any help as allowing them their privacy (22, 4.29%), telling them to keep it a secret as many other people will avoid them (22, 4.29%), advising them to use drugs to calm the mind (18, 3.51%), rallying other friends to cheer them up (75, 14.6%) and I don’t know (16, 3.12%).

The SIPD vignette was answered by 473 participants. The correct answer was chosen by 327 (69.13%) participants. Incorrect choices were: not offering any help as you are allowing them their privacy (10, 2.11%), telling them to keep it a secret as many other people will avoid them (28, 5.92%), advising them to use drugs to calm the mind (36, 7.61%), rallying other friends to cheer them up (33, 6.98%) and I don’t know (39, 8.25%).

The SAD vignette was answered by 446 participants. The correct answer was chosen by 179 (40.13%) participants. Incorrect choices were: not offering any help as you are allowing them their privacy (0, 0%), telling them to keep it a secret as many other people will avoid them (5.61%), advising them to use drugs to calm the mind (22, 4.93%), rallying other friends to cheer them up (187, 41.93%) and I don’t know (33, 7.4%).

### On the comparison between the descriptive statistics of township and an urban school

Regarding the MHL level of township schools compared to the urban school, the participants from the urban school showed better literacy with statistical significance on the following measures: recognition of SIPD, SAD (*p* < 0.0004 and *p* < 0.0001, respectively) and first aid help towards someone with SIPD (*p* < 0.001).

### On the comparison between the ability to recognise a mental disorder and demographic factor

The employment status of parents and guardians was significantly associated with the ability of the learners to identify all the three disorders (*p* < 0.05). For both vignettes of SIPD and SAD, staying with both parents was also significantly associated with the correct identification of disorders. For the vignette of SIPD, learner grade (grade 11s) was significantly associated with the ability to recognise the disorder.

## Discussion

The study sought to understand the MHL pertaining to three mental disorders, namely, MDD, SIPD and SAD among secondary school learners in a region of Tshwane. It also explored whether there is any association between the MHL and the urbanity of the area, as well as association between demographic factors and the level of MHL.

### Mental health literacy about major depressive disorder

Symptoms of depression were recognised by 80.71% of participants. A further 10.35% chose (some kind of) mental illness, giving 91.05% of participants recognising a mental condition.

Previous research indicates a variable ability to recognise depression in similar age groups as the participants in this study.^25^ In some of those studies, recognizing a depressive disorder was much lower than what was found in this study.^26,27,28,29^ However, in other studies, the findings were comparable to this study.^30,31,32,33^ This study shows that although most of the participants could recognise symptoms of depression, close to 19.29% could not. Furthermore, although symptoms of depression were readily identified, the majority did not consider those symptoms as making for a mental illness. Thus, there is scope for mental health education as depressive disorders are considered highly prevalent among adolescents.^23,34^ In addition to the fact that most participants did not consider symptoms as making for a mental illness, it perhaps suggests that campaigns on improving literacy regarding mental issues should start at the point of concertising learners about the physical and the mental aspects of humans. And maybe the emphasis should be on highlighting that the mental aspect of humans is just as important to notice and to ensure it is healthy as the physical.

Specialist mental healthcare practitioners were chosen as the best help for people with depressive symptoms by 52.55% of participants. Another 12.42% chose non-specialist mental healthcare practitioners, bringing the total responses that indicate a professional choice for professional help up to 64.97%. Of note was that though 80.71% correctly identified symptoms as depression, only 64.9% thought that professional help was indicated for the symptoms, suggesting that knowledge regarding symptom recognition did not necessarily translate to knowledge about the best form of help for all the participants. The notable fact that lesser participants, only 12.42%, thought that non-specialists’ mental healthcare practitioners could address the symptoms was very concerning. The concern comes because the first points of medical contact in health regions are non-specialist mental healthcare workers at primary healthcare clinics, who may refer to specialised medical services, including psychiatry. The finding of a preference to choose specialised mental healthcare will also be seen for SIPD and SAD, and consequently, the implications will be discussed here. It may be that participants do not know the lines of referral and the pivotal role that general practitioners play in the rendering of mental healthcare services. That being so, people may also find that approaching a specialised service first will result in being informed to first contact a generalist service. Those seeking mental health services may find this disappointing, because they might expect immediate access to specialised services. This in turn may lead to not seeking professional help at all, meaning that communities need to be educated about levels of health services and lines of referral.

A sizable minority (35.05%) made choices other than a professional mental health service, whether primary healthcare or specialist services. The other choices included family members and friends (24.39%), which is not an uncommon finding in previous studies.^35,36,37,38,39,40,41^ One study even reported on the reason for choosing family and friends, namely that family and friends are already familiar with the person’s background and therefore are an accessible opportunity to share the burden associated with depression.^42^ Nonetheless, it is also possible that depressive symptoms are considered ‘merely stress’ rather than a possible disorder. Turning to lay assistance may be detrimental and even contribute to stigma.^43,44^

Regarding knowledge about psychological first aid, the finding that most participants (63.30%) chose to listen to the symptomatic person in an understanding way and suggest professional help tallies to some degree with the recognition findings, but it is less than what would be expected. Instead, the next most popular choice, rallying friends to cheer them up (14.60%), indicates that the degree of the seriousness of the depressive symptoms is not well understood.

### Mental health literacy about substance-induced psychotic disorder

Symptoms of SIPD were recognised in 61.96% of participants and another 16.86% chose (some kind of) mental illness, giving a total of 78.82% who recognised a serious mental condition. As was the case with participants not choosing that depressive symptoms are indications of a mental disorder, in the SIPD case, few participants indicated the symptoms as those of a mental disorder (16.86%).

Most previous studies that investigated teenagers’ ability to recognise psychotic symptoms focused on just the recognition of psychosis.^45,46^ In this study, the focus was not only on the recognition of psychotic symptoms, but also the ability of participants to recognise the association between psychotic symptoms to the use of cannabis. Being aware of that link is important, because cannabis use, especially at a younger age, has been associated with multiple adverse effects, including psychosis.^47,48^ Moreover, it seems that cannabis use is on the increase,^49,50^ and that cannabis is the most frequently used illicit drug in South Africa.^51^ This indicates the need to raise awareness about SIPD among young people.

Specialist mental healthcare practitioners were chosen by 63.83% of participants to help someone with cannabis-induced substance disorder, which is higher than for depression. Another 25.24% of participants chose non-specialist mental healthcare practitioners (also higher than for the depression vignette) giving a total of 89.07% who chose a professional option for receiving help. That is a higher frequency compared to those who recognised SIPD together with those who simply recognised a mental illness. Only 0.24% of participants thought close friends and family members could help the person in the vignette as opposed to 24.39% in the MDD vignette. Also, in responding to the question about how to assist in such a case, the majority chose (69.13%) that they would listen in an understanding way and suggest professional help. Thus, it seems that the symptoms in the SIPD vignette are more readily seen as serious with a high chance of participants directing such patients in the right direction for help. Nonetheless, 7.61% of participants indicated that they would advise the further use of drugs in order to calm the mind, which would be detrimental.

### Mental health literacy about social anxiety disorder

Symptoms of SAD were recognised by 67.91% of participants. A further 9.45% indicated that there is a mental disorder, giving a total of 77.36%. This is a surprising finding, because previous studies found that psychotic disorders and depressive disorders were better recognised in adult and adolescent populations compared to anxiety disorders.^26,52,53^ In a recent community MHL study in adults in the same region, with the choices between an anxiety disorder, depressive disorder and psychotic disorder, it was also found that the anxiety disorder was recognised the worst.^54^ Thus, the result of this study suggests that the adolescent participants in this study have a higher ability to recognise an anxiety disorder than the adult population in the same region. Of note, however, is that like with the MDD vignette, very few participants choose the option that the symptoms indicate mental problems, which again points towards the need for community mental health education that includes adolescents and adults.

Specialist mental healthcare practitioners were chosen by only 23.86% of participants to help someone with SAD and non-specialist mental healthcare practitioners by 14.70%. This gives a total of 38.56% who chose professional help, clearly much less than for MDD and SIPD. The majority chose close friends and family members (42.17%) as the best source of help. This suggests the need to raise awareness about SAD as well, and perhaps all anxiety disorders. Contrary to MDD and SIPD, fewer participants (187, 41.93%) indicated that listening in an understanding way and suggesting a professional service would be the best help for SAD symptoms. Also, contrary to MDD and SIPD, many more participants (179, 40.13%) indicated that rallying friends to cheer them up was the way to help someone with SAD. This indicates that SAD symptoms are possibly not conceptualised as a mental disorder. Participants may think SAD symptoms are simply a form of shyness, hence rallying friends as a solution. The results suggest that the majority of participants could identify SAD symptoms and even label them as such, but that they lack awareness of the seriousness and the best way to deal with them. This, in turn, calls for mental health education.

### Mental health literacy of urban school compared to township schools

Participants from the urban school performed better with statistical significance only on the following measures: recognition of SIPD (*p* = 0.0004), recognition of SAD (*p* < 0.0001), for SIPD on how to best assist (*p* < 0.001), and for MDD on the best form of help to recommend (*p* = 0.039*). The result is consistent with the findings from other studies.^55,56^ It is most likely because of easier access to information in the urban population compared to the township population. A possible solution is providing education using facilities accessible by all learners (both from urban areas and township areas), for example, at schools, local libraries, or talks by knowledgeable adults considered as role models.

### Mental health literacy and sociodemographic factors

The employment status of parents and guardians was significantly associated with the ability of the learners to identify all the three disorders. This result is similar to that from other studies.^57,58^ This may possibly be accounted for by better socioeconomic conditions in households with employed parents and guardians accompanied by more ready access to information from various media. For SIPD (*p* = 0.017) and SAD (*p* = 0.005), staying with both parents was significantly associated with their correct identification, and for SIPD, learner grade (11s) was significantly associated with ability to recognise MDD (*p* = 0.038). These findings are not reflected by other studies and difficult to explain beyond being isolated findings.

Although short of being statistically significant, male participants identified the case of MDD better than females (0.089), a finding contrary to most studies.^59,60,61^

## Conclusion

Even though the result is promising regarding the recognition of all three disorders, there is still room for improving MHL levels, especially in the area of seeking help and knowledge about anxiety disorders in general.
